# Serial Murder of Four Victims, of Both Genders and Different Ethnicities, by an Ordained Baptist Minister

**DOI:** 10.1155/2011/163403

**Published:** 2011-07-28

**Authors:** James A. Reavis

**Affiliations:** Intrapsychic, 3636 Camino Del Rio North no. 230, San Diego, CA 92108, USA

## Abstract

A case of a 61-year-old African-American male who sexually assaulted and murdered four individuals, of different ethnicities and both genders, is reported. The subject additionally engaged in sexual activity with each victim postmortem. Each murder is reviewed in detail, and the subjective state of the offender during the murders is commented upon. Psychological test data are reviewed. The subject met criteria for several Axis I disorders, including Bipolar I Disorder, Pedophilia, and Sexual Sadism, and met criteria for Axis II diagnoses of Narcissistic and Antisocial Personality disorder. He was additionally classified as a Psychopath, which, in combination with his Sexual Sadism, general psychiatric state, and exquisite sensitivity to humiliation, led to his decision to murder.

## 1. Introduction

A serial murderer was defined by Geberth and Turco [1] as “an individual who, either alone or with a partner, is suspected of killing at least three people, over a period of time with breaks between the murders (p. 54).” These authors found that the majority of subjects whose crimes met this definition (*N* = 387) had some kind of sexual motivation for their killings, partially by virtue of the fact that most of them (248/387) had sexually violated their victims. They additionally reported that every subject for whom diagnostic criteria could reliably be made (*N* = 68) met DSM-IV [2] criteria for both Antisocial Personality disorder and Sexual Sadism. Fully 60 percent of the subjects were under age 30, and, in contrast to lay public beliefs' on the topic, the proportion of African-American subjects (15.8 percent) was slightly greater than the national population average. 

Douglas et al. [3] defined sexual homicide by stating that a “sexual element” was the “basis for the sequence of acts leading to death (p. 123).” Meloy [4] defined the same behavior as “the intentional killing of another human being during which there is sexual activity by the perpetrator (p. 2),” in this review on the subject, the author estimated the rate of sexual homicide at less than 1 percent, based on the proportion of homicides reported to law enforcement per annum. He additionally reported that the perpetrator of a sexual homicide is most often a young man under the age of 30, who kills a female of the same race who is either a stranger or acquaintance of his, as opposed to an intimate partner. With regard to the psychopathology of sexual killers, Meloy also wrote that the vast majority share traits of narcissism and psychopathy, are likely to be diagnosed with Sexual Sadism, and are unlikely to be psychotic at the time of the crime. With regard to the use of substances, other authors [5] have reported that nearly half (*N* = 118) the sexual homicide perpetrators in their study reported use of alcohol prior to the killing, while just over one third reported use of drugs.

In the largest known empirical work on such subjects (*N* = 122), necrophilia, the sexual arousal to human corpses, was differentiated by Rosman and Resnick [6] into two discrete categorizations, labeled genuine and pseudonecrophilia. Genuine necrophilia was further divided into categories of necrophilic homicide, during which the perpetrator commits homicide to obtain a corpse for “special purposes,” regular necrophilia, which comprised the use of corpses for sexual pleasure in the absence of a precipitating homicide, and necrophilic fantasy, in which the individual gives evidence of sexual arousal to corpses only through fantasy paired with masturbation, in the absence of behavioral acts. In contrast, pseudonecrophilia was defined as “transient attraction to a corpse, but corpses are not the object of his sexual fantasies (p. 154-5).”

This paper discusses the sexual homicides of four victims, of both genders and different ethnicities, by a male 61-year-old African-American ordained Baptist Minister, in Los Angeles and San Diego Counties, over an 11-month time period, during the years 2000 and 2001. (The subject has given his full informed consent for the author to disclose all information pertaining to this case and his personal life.) The above brief review on the topics of serial murder, sexual homicide, and necrophilia will be compared with the particulars of this case. The actual acts of the perpetrator and his subjective viewpoint during the murders, in combination with crime scene information and psychological test data, will be utilized to create a comprehensive depiction of the factors that precipitated the decision to murder four human beings. Each homicide will be examined according to three distinct stages: the period during which Mr. D. met the victim and engaged in consensual activity, the period of coercive sexual activity, and Mr. D.'s postmortem sexual activity with his victims. A fourth stage, the disposal of the body, will be covered only briefly. The quotes included are Mr. D.'s *verbatim *statements regarding his subjective experience. (Clinical interview of the subject lasted 19 hours and was videotaped. The entire interview was transcribed *verbatim.*)

## 2. The Offenses

In late May of the year 2000, when he was 59 years of age, Samuel D. met an apparently homeless African-American female, a casual acquaintance of his in her early 20's, on a street near his apartment in Los Angeles. (“Alicia's” body was never found. Mr. D. was convicted of this crime based on his admission to this homicide and three others, forensic details of which corroborated his report.) He “became curious” as to whether he could engage the woman in sexual activity and explicitly denied during interviews subsequent to the murder the existence of coercive or homicidal fantasies at this juncture. The two walked back to Mr. D.'s apartment, showered together, and engaged in consensual fondling. Mr. D. supplied Alicia with alcohol, crack cocaine, and marijuana. He did not use any substance. The two engaged in multiple vaginal intercourse for a period of between 24 and 48 hours, by Mr. D.'s estimate: “there was a period of at least a day or so that she would have been there nude the whole while. And having sex with me … like constantly.”

Consensual sexual activity was brought to an end when Alicia expressed a desire to leave Mr. D.'s apartment. When asked if her refusal to continue to have sex with him was frustrating, he stated, “I didn't like it. If I got mad I wouldn't know that I got mad.” In sequence, Mr. D. reported that the following occurred: he prevented Alicia from exiting his bedroom by blocking the doorway with his body; he “struck her a few times in the face,” to incapacitate her and quiet her struggling; he used stereo speaker wire as ligature to bind the victim's hands and feet behind her back, legs bent at the knee with feet in the air: “I tied her so that her buttock area, her vaginal area would be exposed.” He was questioned on his conscious motivations for binding Alicia. He reported “I don't want her to leave, I want to *keep *her. All I wanted was to fuck, I kept telling her that.” He was also asked whether the act of binding the victim was sexually arousing to him: “I don't remember feeling sexual at all during that time, aroused or anything. The [ligature] was just to like make her still in the position I wanted her in.”

In contrast to his claim, evidence on each victim for which information is available suggests that Mr. D.'s use of either ligature or violence was extreme, and *more* than adequate than that which would be used only for restraint. For example, Mr. D. gagged Alicia with a blouse, sweater or washcloth (He was unable to remember precisely). When she would not quiet, he threatened her with a butcher knife approximately 12 inches long by 1.5 inches wide, by holding it above her chest in a stabbing gesture. He verbally threatened to kill her. Mr. D. was asked if he thought the victim was frightened (at this point she was bound, gagged, and was being threatened with a butcher knife). He replied “I don't think so.” It is possible that the deficit in affect from which Mr. D. suffered, which facilitated both the lack of empathy for others and his use of predatory violence, rendered him unable to recognize the extreme nature of his behavior. It is also possible, however, that his denial of sadistic sexual pleasure is related to his knowledge that the level of violence used in his crimes would lead to a harsher penalty during the sentencing phase of his legal case.

Mr. D. stated that he felt “something akin to anger or frustration” at Alicia's constant struggling. He reported that, for the first time, he thought of drugging Alicia with the sedating tricyclic antidepressant amitriptyline. The medication was in 25 mg tablet form, which he placed in the victim's vagina and rectum. Mr. D. also fed the victim through a straw, into which he had placed medication. When sedation took effect, the victim's struggles quieted. She remained bound and gagged. He now began to anally rape the victim, and when questioned on her emotional state at this point, he reported “she reacted as if it was … extremely painful … [but] I don't know what her reactions mean, I can only guess. They don't give me a clue even now.” 

Mr. D. was asked to report on his subjective state, at the point in time at which he had immobilized his victim and her anus and vagina were exposed for his use. He reported “I could do what I want now. This girl is under my control. There are no barriers, there's no resistance. Unlimited sex is here.” Mr. D. then anally and vaginally raped this victim for a period of approximately 24 hours. He was asked what he felt for Alicia during this time: “nothing, except here's a body.”

Sexual activity ended after approximately 48 hours of consensual sex and 24 hours of coercive sex, when the victim died while being raped. Mr. D. denied any acute action by him that led to the victim's death. In each homicide for which information is available, however, strangulation has been listed as one possible cause. 

Mr. D. continued to have anal and vaginal sex with the corpse, without pause from pre- to postmortem, for a period of roughly 48 hours. The corpse remained on his bed, bound and gagged. He covered the victim's head with a cloth he found in his room, secondary to revulsion at the sight of her face, which was bloated and swollen from his blows. He was asked what was specifically arousing to him regarding sexual intercourse with a corpse. He responded “It was just here's a hole, basically.” For the first time since her arrival, Mr. D. climaxed and ejaculated inside the victim's vagina. 

After roughly two days of nearly continuous sex with the corpse, Mr. D. “couldn't stand the smell or sight” of the body anymore, and carried it from the bed to the bathtub. He reported that he had read a book on Jeffrey Dahmer and developed the idea to dispose of the body by pouring onto it bleach, liquid Plumr. lye, drano, and other solvents, in attempts at “dissolving it.” The body remained in the tub during the events of the second homicide.

Approximately one week after he had murdered Alicia, Robyn W., a 37-year-old, African-American female acquaintance of Mr. D. appeared alone and unannounced at his door. Mr. D. quickly engaged Robyn in sexual conversation, and she agreed to enter his apartment. 

Consensual sexual activity consisted of multiple vaginal and anal intercourse, for a period of between 24 and 48 hours. As he had done with Alicia, Mr. D. bought the victim crack cocaine, which she smoked at his apartment, and Cisco wine; once again, he did not use substances. Robyn's statement to Mr. D. regarding her desire to leave the apartment to care for her children, signaled the onset of coercive activity. He “pinned” Robyn to the bed by sitting on her chest, and then bound her in a fetal position, so that he could “get at … either her pussy or her butt …where I could stand up on the bed and fuck her with her facing up, on her back, with her feet and knees in the air. (The defendant placed pillows underneath Robyn so that he could have easier access to her vagina and anus as he stood on the bed.)” When asked of his subjective experience at this point, Mr. D. stated “I have control of them now. All the tying and drugging had achieved was to let me have sex like uninterrupted without … fighting, because … that detracted from it for me.” Once again, Mr. D. denied sadistic arousal from the victim's pain. The specifics of the behavioral evidence, however, suggest otherwise. 

Robyn began to plead with Mr. D. and mentioned that she had children. Mr. D. reported that he “ignored it,” though may have said to her “I told you what I wanted.” He threatened her with the same knife he had used with Alicia. After he had restrained her with ligature, he gagged Robyn with a sweater he found on his bed, which was fastened into place with wire. He pulled another sweater over her head, covering her entire head and mouth. (This action was likely motivated by twin desires: to denigrate the victim and to heighten his own sense of control.) In contrast to his actions with Alicia, perhaps because he remembered the struggle which had ensued, Mr. D. immediately drugged Robyn with amitriptyline by inserting two to three tablets into her vagina. He then anally raped this victim—who remained bound, gagged, and drugged—for a period of between 36 and 48 hours, by his estimate. He denied any conversation: “there was no reason for conversation … there was no way she could stop it.” After carrying Robyn to a corner of his bedroom and covering her with clothes and linens, Mr. D. left the bedroom for his kitchen, where he remained for between 15 and 60 minutes. He returned to the bedroom intent on continuing to have sex with Robyn, but reported that she was dead upon his entrance to the room.

The coroner's report of this death stated that death occurred from “apparent multiple ligature strangulation,” and “possible blunt force trauma to the head and face.” Mr. D. stated to two investigators and to myself that Robyn died while he was away from the room. He stated during a separate psychological evaluation that Robyn died during the act of being anally raped. Although he denies it, medical evidence suggests that Robyn died secondary to a volitional act from Mr. D.: either manual strangulation or blunt force trauma to the head.

Mr. D. reported that “the fact that [Robyn] wasn't physically responding was not a problem.” He did not remove the ligature from Robyn postmortem and stated that as he continued to have sex with the body he did not want to ejaculate because “climaxing ended the sex. I [would have] had to stop. It was just sex, just sex, just sex and I didn't want it to end.” Mr. D. remains uncertain whether he ejaculated inside the corpse of Robyn. After roughly two days of nearly continuous vaginal and anal sex with Robyn's corpse, Mr. D. “pushed” the body of his second victim into the bathtub, on top of his first victim. Like in the first homicide, the odor of the decomposing body, and the lividity change, precipitated his decision to discontinue sex.

Mr. D. stated that during the time period between the death of Robyn and the death of his next victim, he began to consider abducting a person to keep as his “sexual slave.” He considered keeping the person either in a “hiding place” outside his apartment, and then transporting the victim to his home for sex, or inside the organ he had at his apartment. Around this time, he wrote a short narrative which makes clear that, while the death of Alicia may have been “accidental,” as he claimed during investigation after his confession, his subsequent behavior was planned, purposeful, and predatory. Mr. D. wrote: “tools of the trade—cable wire, clothes, necktie (regarding his use of ligature, Mr. D. stated to me that he would use “anything that was nearby … like a necktie.” This statement appears to belie that suggestion; it appears that ligature was chosen before the crimes, for specific purposes), gag-rope (and the search for more), better gag, then stocking cap and later, wire mask to keep in on. c∗r got them off several times. (Why didn't they scream?-fear of drugs. Need hand and leg cuffs—too many escapes. (learning the ropes) getting them on (g kept gedding loose and r same flysr kept waking up and moving things as if she knew what was coming …”

Either accidentally or deliberately, Mr. D. found an activity that suited his sexual appetites. He had no internal prohibitions in the form of conscience or fear of punishment which would preclude drugging, bondage, and rape. During our interview, Mr. D. took exception to my use of the term “serial killer” to describe him self. In responding to me that although he did not “see” himself as a serial killer, he stated that he might have “killed more had the opportunity or circumstances been different.” After he had committed his first murder, Mr. D. decided to kill again by using similar methods.

Approximately 7 to 14 days after he had killed Robyn, Mr. D. met his next victim, Salvador, a mentally disabled Hispanic male approximately 36 years of age. Mr. Reyes, according to Mr. D., approached him and offered sex in exchange for $15. Mr. D. accepted and the two walked from the street to Mr. D.'s apartment.

Consensual sexual activity consisted of multiple anal intercourse for a period of roughly four hours. Mr. D. assumed the dominant “top” position and did not allow Mr. Reyes to penetrate his anus. They did not kiss. (Kissing is perhaps the most affectionate sexual activity. Mr. D. either kissed his victims before they were bound or drugged, as a predatory “grooming” behavior, or did not kiss them at all.) During this intercourse, Mr. D. decided that Salvador “was not ever going to leave … I wanted to keep him there with me.” He equated Salvador to a “sex toy” and to a “sexual slave.”

Mr. D. drugged Salvador by giving him a beer into which he had placed amitriptyline. He also inserted tablets into Salvador's anus. He then gagged him and put a nylon stocking over his head. Salvador's struggles irritated Mr. D., and the two “began fighting.” Mr. D. again denied being sexually aroused by the act of binding the victim, by his pleas, or by the obvious pain the victim was in. He anally raped Salvador for approximately two days and one night, by his estimate. During times when Mr. D. left the apartment when Salvador was still alive, he carried him by the ligature to a closet. Upon his return, he transported Salvador back to his bed, to continue to rape him. 

Salvador died while being anally raped. Mr. D. was asked what he had done to precipitate Salvador's death. He responded “nothing I knew except just fucking him.” Mr. D. did not pause at the time of death, but “went on” anally raping Salvador, and continued to do so for between 24 and 48 hours, by his estimate. He was asked if it was more arousing to have sex with a live person or with a corpse. He stated “it didn't make any difference. I don't remember it being any different.” Mr. D. continued to engage in sexual activity with the corpse of Salvador until the body began to smell, at which point he put the still-bound body into a plastic bag, carried it down the stairs and placed it into a shopping cart, which he used to transport the body to a local park.

On Saturday, 28 April 2001, approximately 11 months after he had committed his first homicide, Mr. D. traveled to San Diego to take part in a poetry reading at a local book store. He and his male companion arrived too late for the reading and were rescheduled; Mr. D. chose to spend the rest of his night in San Diego at a bath house frequented by gay males, for the purpose of engaging in anonymous sexual activity.

Soon after his arrival at the bath house, he attempted to place several amitriptyline tablets he had brought with him from Los Angeles in the anus of a sleeping Black male; this man awoke briefly and pulled the sheet on which he was laying tighter around his body, which precluded Mr. D. from drugging him. He next engaged in simultaneous sexual activity with three men. During this, Gary B., a 46-year-old gay Caucasian male stranger approached him and expressed a desire for sex with the defendant, alone. Mr. D. refused, though after he had completed the sexual act with the other males left to find Gary. 

The two engaged in consensual anal intercourse at the bath house, after which Gary invited Mr. D. to his apartment in San Diego. They arrived at the apartment in the early morning hours of Sunday 29 April. Although Mr. D. had planned to drug Gary's food early during this time, he did not because Gary did not eat with him. They again engaged in multiple consensual anal intercourse for a duration of several hours. They parted company during the day of Sunday and met that evening at Gary's apartment, where Mr. D. watched pornographic videos. 

At approximately 5 P.M. the two engaged in multiple anal intercourse for a period of several hours, during which Mr. D. drugged Gary with amitriptyline and diphenhydramine. The method of drugging, in contrast to his activity with previous victims, was to insert the tablets into the foreskin of his penis, then insert his penis into Gary's rectum. After a long period of sexual activity, Gary informed Mr. D. that he wished to discontinue the sexual activity and wanted to sleep so that he could go to work that morning. Mr. D. unplugged the clock in Gary's room to prevent him from knowing the time. He later told investigators that the victim's statement regarding his desire to stop having sex “irritated” him. He got up from the bed and, in a sullen gesture, went into the living room. Gary appealed to Mr. D. to either come back to bed and sleep or leave his apartment.

Mr. D. was angry at Gary for not giving him what he wanted, and felt rejected and insulted by his suggestion that he leave. He stated to investigators that Gary's statement, “set [him] off … I was upset he didn't let me do what I wanted to do.” Gary, said Mr. D., “should have let me do what I needed to do. (Mr. D.'s statement to investigators is similar to his statement to Robyn, during the time she was bound, gagged, and fighting him: “why you fighting me? I told you what I wanted.” The absence of empathy, and presence of entitlement, leave Mr. D. irritated and perplexed by his victim's actions.)” Mr. D. next returned to the bed and attempted to insert his penis into the sleeping Gary's rectum. He recognized that Gary was “trying to let [him] know” that he wanted to sleep, which further angered him. Mr. D. successfully inserted his penis, however, and the two had anal intercourse for between 30 and 35 minutes.

As Gary's resistance weakened, Mr. D. “took over and did what [he] wanted to do,” which was to anally rape him. Gary again began to struggle, and Mr. D. attempted to bind him with ligature. He used a plastic coated wire cord to tie one of Gary's ankles to the bed. He used shoestrings and attempted to tie his hands together, but Gary was able to struggle free. At this point, Gary began to scream, whereupon Mr. D. attempted to gag him with a dark blue towel. He then left the bedroom and went to the kitchen, where he found an electric “bread dough mixer,” similar to a rolling pin. He returned to the bedroom and struck Mr. B. twice in the head with this instrument, intending to “knock him out.” From behind, Mr. D. grabbed Gary around his neck, with his right arm, and shoved large amounts of petroleum jelly into his nose and mouth to quiet his screams.

Mr. D. reported to police investigators that Gary died during this struggle, after he had “pulled up” on his mouth with his left hand. The coroner's report stated cause of death as asphyxia, either from “forcible covering of the mouth” or from “pressure on the neck.”

Regarding Gary's death, Mr. D. stated “he just slumped over and stopped moving. I … decided he must be dead. Then I went back and tried to put him on the bed so I could have more sex with him.”

Mr. D. used ligature to bind the body in a fetal position postmortem, to aid his attempt at placing Gary's body back on the bed, so that he could have anal intercourse with the corpse. However, he soon discontinued his attempt, after coworkers called Gary's house to check on his whereabouts, and instead dragged the body into a bedroom closet. He filled four plastic trash bags with the victim's belongings, unplugged the telephone, and attempted to carry the bags from the apartment. The bags broke; Mr. D. then took “what [he] thought might have [his] name on it,” and Gary's checkbook and apartment and car keys and exited the apartment.

Several days after killing Gary, Mr. D. returned to San Diego by bus, intent on “taking off”—robbing—the apartment. He saw police tape across the door frame of the apartment and thought better of entering. He did, however, use the victim's car keys to steal his vehicle and drove back to Los Angeles.

On 17 May 2001, Mr. D. was arrested in Los Angeles for the theft of Gary's vehicle. On 01 August, while in custody in San Diego after being charged with auto theft (Mr. D. was originally charged with auto theft and murder; the latter charge had been dropped at the time of his confession), Mr. D. approached a correctional counselor and stated “you're going to go down in history. God is telling me to tell you [of the four murders]” he had committed.

### 2.1. Living Victims

Mr. D. also left several living victims, whom he either attempted to drug, rape, or kill. Several days after killing his second victim, in June 2000, Mr. D. resumed sexual encounters with a “light skinned” Black male in his early 20's. The two had engaged in sexual intercourse on several occasions in the recent weeks, either at Mr. D.'s apartment or at a local park in Los Angeles. After Robyn's death, Mr. D. attempted to drug this man by encouraging him to drink beer into which he had dissolved amitriptyline and by attempting to insert tablets of the medication into his rectum. He reported that although the man drank the beer, he did not become sedated to the point that he could do as he hoped, which was to bind and rape him. When asked whether he would have killed him, Mr. D. reported that he “probably” would have tried to “keep him.”

During the same time period, between the time of Robyn's death and the death of Salvador, Mr. D. also drugged a longtime friend of his, Michael T., with whom he had often engaged in consensual anal intercourse, by giving him drug-tainted beer. However, Michael may have been using crack cocaine at the time and sedation did not take effect. If it had, Mr. D. reported that he would have bound and raped Michael.

Mr. D. had originally met Robyn through a young Black male named Tyrone B., who, according to Mr. D., was either her boyfriend or pimp. During the summer of 2000, prior to Robyn's death, Tyrone broke into Mr. D.'s apartment while he was sleeping. It soon became clear to Mr. D. that Tyrone was going to use his apartment as a “crash pad,” and had no intention of leaving. In response to this, for a period of one week Mr. D. put amitriptyline in Tyrone's Kool Aid and into the food he gave him. He did not have either consensual or coercive intercourse with Tyrone.

In June or July 2000, in the weeks after he had disposed of Salvador's body, Mr. D. had sex with two Black females, one of whom came to his apartment on several occasions, whom he did not attempt to drug or bind. A third Black female, who refused to have sex with him, also visited his home during this time period. While she was sleeping in his bedroom, Mr. D. dressed in overalls and a woman's white fur coat. He pulled “greenish” colored netting over his face and fastened it around his neck. He snuck into the bedroom, intent on binding her with the ligature he had recently begun to collect. Mr. D. accidentally caused a television set to fall over, which caused the woman to stir in bed. Mr. D. “raced” out of the room, removed the disguise (“I [could] say to her, someone was in here, someone tied you up.”) and then reentered the bedroom. He reported that he did not speak to the women and she did not question him regarding who had been in the bedroom. Mr. D. denied ever successfully binding this woman and stated that she simply “walked away.”

In the days before the murder of Salvador, in June 2000, and continuing for a period of several months, Mr. D. drugged his lover of four years, Tom B. He reported that “things about [Tom] were constantly perturbing” him, including his belief that Tom continued to grieve the death of his former lover (“face reality, he's dead. What's wrong with me? I'm alive”), that he controlled the amount of time the two spent together, and that he controlled the type of sexual activity the two engaged in. All of these were felt by Mr. D. as humiliating and gave rise to deep resentment. Mr. D. reported that he had reached a point at which he was “not only trying to be dominant over [Tom], but run his life.” He told Tom that what God wanted was for him (Tom) to be his lover.

After the murder of Salvador, Mr. D. began to drug Tom's food, by crushing medication in a small bowl and putting it in food he gave him, then by putting it in boxes of cereal, in milk, ice cream and cream for the coffee, and in the ice Tom used in his drinks. He reported that his goal was to keep Tom constantly drugged, so that he could have sex with him whenever he came to the house. During an argument between the two over the telephone, Mr. D. told Tom that he had killed three people, something which was motivated by a desire to “make [Tom] obey” him by frightening him into submission. Mr. D. told Tom that he was continuing to kill and at one point told him that he had murdered 30 people. Regarding this strategy, Mr. D. stated, “this makes him listen to me.”

Mr. D. also considered killing Tom while he slept. He stood above him and thought of twisting a stereo wire around his neck, of striking him in the head with a hammer, and of using ligature to tie him to the bed and killing him there. He denied ever actually attempting any of these. After roughly two months of drugging Tom, Mr. D. told him of his actions. He stated to him “I didn't want to kill you. I didn't want to drug you, but you're making me do it. If you listen to me and do what I ask and don't make me mad … I try to tell you how to get along with me, and you won't do it. I don't want to hurt you. I care for you.” After this, the two apparently reconciled. Soon thereafter, Mr. D. became embittered by a perceived slight from Tom and again began drugging him. This time, Tom recognized what was being done and confronted Mr. D., who again told Tom that if he would simply do as he was asked, he would not have to resort to such measures.

## 3. Background Information

Mr. D. was born and raised in Wilmington, NC, USA, in 1940, one of six children born to a Baptist Minister father and a homemaker mother. He denied any history of known developmental problems or delays, denied a history of physical or sexual abuse, and denied any juvenile history of arrest or involvement with social services. He described excellent academic achievement and ultimately graduated from a small liberal arts college in Ohio, in 1962.

His psychiatric history is positive for episodes of both depression and mania, during which he would go without sleep, suffer delusions, hallucinations, racing thoughts, grandiosity, and massive agitation. For example, after he had committed his first three murders, Mr. D. traveled to Little Rock, Ark, USA, where he heard the voice of God telling him to “herald the end of the world.” He was taken to a psychiatric hospital for a brief period and released without antipsychotic medications. Mr. D. denied, however, active symptoms of either mania or psychosis during the time period of the murders.

Mr. D. freely reported that he had “conned and hustled” all his life, beginning with thefts as a child. His first arrest occurred in 1962, at age 22, when, apparently by accident, he struck another vehicle with his own and killed its occupant. He was charged with second-degree manslaughter and sentenced to six months in jail. In 1963, Mr. D. was arrested for burglary and breaking and entering, which involved thefts from a local high school. In 1968, he was arrested for manslaughter, second-degree burglary, and larceny, in 1976 for grand theft, in 1985 for alien smuggling, in 1990, twice, for disorderly conduct and soliciting a lewd act, and in 1997 for assault with a deadly weapon.

Mr. D. also reported a prolific and deviant sexual history, with an onset beginning prior to his adolescence. Specific behaviors included exposing himself, public masturbation, fetishism (involving the insertion of objects into his rectum, such as broom handles, carrots, zucchini, water hose, stool leg), and frottage of children and adults of both sexes, as well as staring at the genitals of various animals, including horses. He additionally reported extensive sexual activity with the following animals: chickens, mules, goats, pigs, dogs, cows, and cats. (I was somewhat incredulous regarding Mr. D.'s report of sex with animals. However, the specifics of his report appear to lay to rest any doubt. For example, he reported that he disliked penetrating goats because, unlike other animals, the fecal matter inside the anus was hard and crusty and chafed his penis.) He admitted to six previous victims of child molestation, all adolescent males, and to professional sexual misconduct, which involved sexual intercourse with parishioners. He additionally reported massive use of prostitutes, sexual affairs, and anonymous sexual activity with strangers.

## 4. Clinical Findings

Mr. D. was neuropsychologically evaluated in custody while awaiting trial, nine months after his last murder. He was interviewed for the first time by myself, in custody, 19 months after his last murder. At the time of our interview, Mr. D., a 62-year-old divorced African-American male, stood approximately 5 ft. 8 in. tall and weighed 150 pounds. He had been disabled for several years, secondary to trauma symptoms subsequent to being assaulted by two males in a Los Angeles area park, in approximately 1985, and for which he was prescribed amitriptyline. Mr. D. also worked intermittently as an ordained minister. He spoke only English and was right-hand dominant. Finally, he had contracted the HIV virus in the early 1980's, though had never been prescribed medication for his disease.

Mr. D. produced a verbal WAIS-III IQ score of 117, a performance IQ of 109, and a full-scale IQ of 114, placing him in the 82nd percentile. An extensive neuropsychological test battery indicated that Mr. D. suffered from deficits in executive measures that were predominantly visual, rather than verbal, and exhibited moderate impairment in tests of manual dexterity and visual-motor coordination. Test findings were consistent with an impression of right brain hemisphere impairment, which was likely associated either with dementia stemming from his HIV disease or the increased energy (and associated disorganization) associated with his symptoms of mania.

Mr. D.'s Minnesota Multiphasic Personality Inventory-II profile (MMPI-II), completed in custody after his admission to the murders, produced a valid profile with clinical elevations (*T* > 65), or near-elevations, on scale 5 (*T* = 64), scale 8 (*T* = 65), and scale 9 (*T* = 72). Three supplemental scale scores are also worth mentioning: overcontrolled hostility (*T* = 79), ego strength (*T* = 24), and PK (PTSD) (*T* = 68). The clinical picture wrought from these test scores is of an individual bothered by a past traumatic experience, with strong feelings of worthlessness, distortions in his reality testing, and massive levels of energy and internalized anger. The computer-generated profile interpretation indicated, in part, “a borderline psychotic panic or possibly a psychotic decompensation … his sexual identity as well as his recent sexual activities are likely to be mixed and confused if not odd or peculiar … sexual frustrations are more likely to relate to his conflicts around being self-assertive more than to inhibitions around sexual difficulties … there is a basic conflict between wanting reassurance and protection versus his inability to tolerate a submissive and vulnerable position, so that attempts to establish emotional closeness are repeatedly self-defeating.”

Millon Clinical Multiaxial Inventory-III profile (MCMI-III), completed during my evaluation of him, was also valid and suggested Axis I diagnoses of Bipolar Disorder, manic, severe, without psychotic features (BR 81) and adjustment disorder with mixed anxiety and depressed mood. Axis II diagnoses were suggested in the areas of self-defeating personality traits (“Masochistic” scale BR 82), dependent personality traits (BR 80), depressive personality features (BR 79), and Antisocial Personality features (BR 76). The computer-generated report indicated that Mr. D. was “characteristically dependent and depressed, leading him to anxiously seek reassurance from others and to fear separation from those who provide support” and was caught in “a struggle between dependent acquiescence and inducing guilt in others over their indifference and abuse.”

Mr. D. also produced a valid, though constricted (*R* = 17) Rorschach. These test data indicated that he was highly defended against his own affect, primarily through the use of intellectualization. Also indicated were behavioral passivity and acquiescence to the wishes of others. Consistent with the findings from the measures above were indications of felt inadequacy and worthlessness. The interpretive report indicated that Mr. D. evidenced “marked tendencies to overvalue his personal worth and to become preoccupied with his own needs at the expense of concerns about the needs of others. Likely to exhibit a sense of entitlement; a compensatory narcissism in which he clings to a sense of superiority as a way of defending against underlying feelings of inferiority.”

Mr. D. was also evaluated with the Psychopathy Checklist-Revised (Hare, 1991), to determine his level of psychopathy. His overall score of 33 was well over the recommended cutoff score of 30 for classification as a psychopath and fell in the 90.9th percentile in relation to other male prisoners from the normative sample. His score of 15 on Factor 1 of the test, which comprises items evaluating the selfish, callous use of other people, fell in the 97.6th percentile, and his score of 14 on Factor 2, which is a measure of chronic social deviance, fell at the 71.1st percentile.

In summary, objective and projective tests of personality indicated strong feelings of inadequacy and dependency on others, which were themselves felt as humiliating by Mr. D. and which were defended against by narcissism, intermittent use of passive-aggressive, sullen, and sulky behaviors, and, for just under a period of one year, by sexual domination and murder. The unifying construct in this case, one that allows us to bring together the disparate findings of felt inadequacy, compensatory mechanisms against this, and, ultimately, murder, is psychopathy, as the absence of conscience in Mr. D. allowed him to “feed” his grandiosity through the most extreme behavior imaginable.

## 5. Diagnosis

In light of all the available evidence, the following DSM-IVTR psychodiagnosis is provided in the case of Mr. D.:

Axis I: 296.43 Bipolar I Disorder, most recent episode manic, severe without psychotic feature,
294.1 dementia due to HIV disease,
297.1 delusional disorder grandiose type,
302.2 Pedophilia, sexually attracted to males, nonexclusive type,
302.84 Sexual Sadism,
302.82 Voyeurism,
302.9 Paraphilia not otherwise specified (including the categories of: Necrophilia, Partialism, Zoophilia, Sexual Aggression toward nonconsenting partners,)
309.28 adjustment disorder, chronic, with mixed anxiety and depressed mood,
Axis II: Antisocial Personality disorder,
Narcissistic Personality disorder,
Psychopathic Personality —severe (not included as a discrete diagnostic category in DSM system,)
Axis III: HIV+,
Axis IV: problems related to interaction with legal system/crime,
Axis V: 40: major impairment in several areas of functioning.

## 6. Comparison with Research Findings

This paper began with a brief review of the research into the areas of serial murder, sexual homicide, and necrophilia. Application of those findings to the present case indicates the following: Mr. D. appears to be very similar to the modal serial sexual homicide perpetrator. With regard to Geberth and Turco's [1] findings on serial murder, Mr. D.'s acts meet the definition of serial murder, as he killed at least three victims with a break between the murders. There was a clear sexual motivation in each of his killings, and he meets DSM-IVTR criteria for both Antisocial Personality disorder and Sexual Sadism. He was not, however, under age 30 at the onset of the murders. Although Mr. D. did not only kill female victims within his own race, he does fit with other general findings on sexual homicide perpetrators, as reported by Meloy [4]: his victims were all strangers or acquaintances to him, his personality was marked by high levels of narcissism and psychopathy, and he was diagnosed with Sexual Sadism. Finally, in contrast to the findings of other authors [5], Mr. D. did not use either alcohol or drugs to disinhibit him prior to the killings, which may be a function of his level of psychopathy: there was no felt need to disinhibit himself because he felt no empathy for his victims and was not particularly troubled by his actions. With regard to the findings on necrophilia [6], Mr. D.'s sadistic motivational set and his transient (as opposed to chronic) attraction to corpses fit well with the authors' “pseudonecrophilia” category.

## 7. Psychodynamics and Motivations

The interpersonal and emotional functioning of Mr. D. is, in many ways, similar to that of a child between the ages of one and two. (What discriminates the young child from Mr. D. is the child's capacity to affectively bond to others and the existence of the rudiments of conscience.) When Mr. D.'s Narcissistic needs are gratified, he is satisfied and pleasant. Upon being frustrated or displeased, he becomes rageful and will use whatever means necessary to regain the lost sense of his power. Like a child—and the criminal psychopath—his internal resources and explicit behavior are completely self-focused. He does not have a true appreciation, which occurs through the development of empathy, of others as human beings with their own wants, needs, and desires. Mr. D. continually asked his victims, while they were bound, gagged, and drugged, why they were “fighting” him, as he had already told them “what he wanted.” In answering the question “how do you *feel* about the crimes you committed,” Mr. D. gave a prototypically Psychopathic response: “I want to know why they occurred.” His long history of Antisocial behavior, as well as his actions in the instant offense, can be rationalized as behaviors he was justified in exhibiting in attempting to get what he wanted.

With regard to his self-perceptions, Mr. D. compares himself unfavorably to others and is envious of traits he sees others possess that he does not. He is at once dependent on others for his internal sense of worth and humiliated and ashamed by this felt dependency. These feelings in turn give rise to deep resentment and antagonism. Fearful of being rebuked should he express this anger, he suppresses it, which serves only to increase its intensity. An example of this occurred during an interview this examiner had with Mr. D. At one point in the discussion, I stopped the conversation and made a frank statement to him regarding his answers to questions, which were rambling and tangential. It was an assertive comment which he interpreted as a personal snub. Mr. D. seemed to withdraw into himself, lost eye contact, and became quiet and sullen. He was *ruminating* on the hurt caused him. The interview continued. Shortly thereafter, Mr. D. made a comment about how I just did not understand him. He also stated that although he liked both his appointed attorneys, he most preferred the psychologist who had evaluated him in early 2001, previous to myself. In telling me this, Mr. D. paused, again made eye contact, and stated “Dr. C. told me he was fascinated by me.”

The idea of others' interest in him, of being supremely talented and special (Mr. D. has written letters to several world leaders, offering his assistance in world problems), can be called defensive, or compensatory, narcissism. Mr. D.'s choice of occupation (he is a Baptist Minister) and his preference for the “top” or dominant sexual position, his long history of theft, which provides him with evidence of his own superiority over those who would catch him, his belief that he is God's instrument to signal the end of the world (also a sign of a grandiose delusional system), his public poetry readings, and his vituperative attacks against two landlords who attempted to evict him are additional signs of this kind of defensive attempt to avoid a felt sense of inferiority. Finally, although a biological predisposition for such likely exists, Mr. D.'s symptoms of mania, including lack of sleep, excitability, sexual binges, and feelings of invulnerability, are also defensive methods of coping.

Mr. D.'s motivations for attempting to drug Michael and Tom were different from those which drove his behavior with the other victims. Defensive reactions to felt humiliation, through fantasies of punishment or sarcasm, for example, are not atypical in human beings. The means by which Mr. D. exacted his revenge, however, are unusual. Two dynamics allowed for the behavioral manifestation of what most would experience only in fantasy: sensitivity to rejection and an absence of conscience. Mr. D.'s behavior with both Michael and Tom was driven, in part, by feelings of humiliation, which he had tolerated for the length of his relationships with these men. For example, his sexual encounters with Michael were almost invariably characterized by Michael assuming the dominant sexual position, and “fucking” Mr. D., to use his word. He stated, “Michael was always dominating over me sexually … like, I'm, I'm a female, a feminine, feminine male. (In an autobiography written as part of his criminal defense, Mr. D. wrote of a childhood experience in which his peers called him a “sissy.” The defendant wrote that this experience was “the most hurtful thing [he'd] ever found at school,” and that he “cried about it for months.” Being placed in the “feminine” sexual position by another male likely awakened old feelings of humiliation.)” When asked if he would have gotten pleasure from the act of binding and raping Michael, Mr. D. stated “yes, because of my hatred of Michael. I [would be] top dog so to speak.”

In addition to feeling humiliated by always having to assume the submissive sexual position, Mr. D. also told me that he was physically afraid of Michael. Mr. D. did not tolerate well either feelings of submissiveness or fear. The usual method of defense of these character types is to rid themselves of intolerable emotions by engendering pain, or a similar emotion, in someone else. When Tyrone was drugged and asleep on Mr. D.'s couch, in plain view of Michael, Mr. D. was offered this opportunity. He told Michael that Tyrone had “been there like that for a week or so … I joked with Michael, ‘see I told you.' I'm trying to feel like the man.”

Mr. D. drugged Tyrone when he came to his apartment, though chose not to rape him. He was asked why he did not take sexual advantage of Tyrone after he had drugged him. He reported that he had been offended by Tyrone's statement to him, which indicated that he thought Mr. D. invited people to his home to play cards only to try to engage them in sexual activity. He reported, “OK, you (Tyrone) think that I have guys over here to fuck them … give them money and everything to fuck them. But I haven't fucked *you*.” In this case, having sex with Tyrone would have been felt as a *capitulation*, as confirmation of Tyrone's belief regarding Mr. D.'s predatory behavior with his card-playing partners. It was more gratifying to him to simply drug Tyrone, allow him to sleep on the couch, and *resist* his own desires for sex. Mr. D. reported that after drugging Tyrone for one week, he stopped doing so; the medication wore off and Tyrone walked out of the apartment. The two did not see each other again. 

It appears possible that Mr. D.'s first victim, Alicia, served as both a learning trial and template for his subsequent killings. The quieting of Alicia, by binding and drugging her, may have begun a process of negative reinforcement, wherein the aversive stimulus (struggling) was removed, leading to an increased likelihood that Mr. D.'s behavior would repeat itself. Mr. D. reported to me and to others that he had no intention of killing his first victim; she may have died by drug overdose, by heart attack, or by some combination of the effects of being bound, drugged, and raped. Alternately, at some point, the victim's behavior may have engendered rage in Mr. D., wherein he would have switched *modes* of aggression, from the predatory mode that predominated, to a more emotionally based and rageful (“how *dare* you?”) affective mode of aggression. If this in fact occurred, Mr. D. may have killed the victim in such a state and then returned to his more calculated predation. Although he has stated that each homicide left him “surprised,” it is more likely that the death of Mr. D.'s first victim and its reinforcing effects led him to seek out other human beings with whom he could act in a similar fashion.

Mr. D.'s postmortem sexual activity with his victims was not motivated by true sexual interest in corpses. Rather, his actions are those of a sexually driven psychopath—a person unable to see others as whole human beings. The deaths of his victims gave Mr. D. almost no pause; the intellectual recognition of death was not linked to any internal sensation, such as remorse, guilt, or fear. In fact, it is likely that living, the victims were no more appreciated by Mr. D. than they were dead. He strove to make his victims objects for sexual gratification—crudely put, “holes,” while living; when dead, they remained “holes,” though ones that would not resist, cry, plead, or beg for their lives. In this case, Mr. D.'s sexual behavior with corpses fits well with Rosman and Resnick's [6] conception of a pseudonecrophilic subject group. (Although a diagnosis of Necrophilia is given secondary to the occurrence of necrophilic behavior over a six-month time period.) Finally, powerful psychological reinforcement—the antidote to his gnawing feelings of inadequacy—arose from the defendant's awareness of the victim's helplessness and death. 

Mr. D.'s psychiatric state during the killings was characterized by mania associated with Bipolar Disorder—evidenced by lack of sleep, delusions that he was God's instrument to signal the end of the world, and irritability—a paucity of affectively toned emotions, criminal predation, and sexual interest in and arousal to the pain of others. These symptoms, apart from sexual interest in pain, were chronic in nature. The predatory mode of aggression utilized by Mr. D. was a means to an end—gaining sexual pleasure. The victims' desire to stop engaging in consensual sex and their constant struggling may have produced some amount of irritation and annoyance. However, the binding and drugging of the victims and the free access to their bodies that this allowed him produced feelings of power—likely of omnipotence—in Mr. D. He had at once devalued another person and proven his own superiority. The postmortem sexual activity, while providing additional sexual release, likewise was both a final insult to his victim and perhaps the utmost example of his power: he had killed and could continue to use the corpse, at his leisure, to satisfy himself.

## References

[1] Geberth VJ, Turco RN (1997). Antisocial personality disorder, sexual sadism, malignant narcissism, and serial murder. *Journal of Forensic Sciences*.

[2] American Psychiatric Association (1994). *Diagnostic and Statistical Manual of Mental Disorders*.

[3] Douglas J, Burgess AW, Burgess AG, Ressler R (1992). *Crime Classification Manual*.

[4] Meloy JR (2000). The nature and dynamics of sexual homicide: an integrative review. *Aggression and Violent Behavior*.

[5] Burgess A, Hartman C, Ressler R, Douglas J, McCormack A (1986). Sexual homicide: a motivational model. *Journal of Interpersonal Violence*.

[6] Rosman JP, Resnick PJ (1989). Sexual attraction to corpses: a psychiatric review of necrophilia. *Bulletin of the American Academy of Psychiatry and the Law*.

